# Feasibility and acceptance of self-applied home type-II PSG studies with a patch-based device

**DOI:** 10.1007/s11325-025-03503-z

**Published:** 2025-10-28

**Authors:** Matthew Uhles, Sabina Alisic, Andrea Brown, Robert Doekel, Wes Booth, Joseph Ojile

**Affiliations:** 1 Clayton Sleep Institute, St. Louis, MO USA; 2 Saint Louis University, St. Louis, MO USA; 3 ABio Clinical Research Partners, Midlothian, VA USA; 4 The Sleep Disorders Center of Alabama, Birmingham, AL USA

**Keywords:** Home PSG, Type-II PSG, Self-applied PSG, Usability, Satisfaction, Success rate

## Abstract

**Purpose:**

Onera Health has developed the first wireless, patch-based type-II polysomnography (PSG) system, the Onera Sleep Test System (STS) 1 for self-applied, home sleep studies. This multicenter observational study aimed to assess the success rate of the device in a home setting and gather participant feedback.

**Methods:**

41 patients (age: 48.7 ± 16.9, 58.7% male) with a suspected sleep disorder referred for a PSG were included in the study. Participants received minimal instruction at the clinic and were mailed the Onera STS 1 with instructions to complete a home sleep study. After completion, they returned the device by mail. At the fulfilment center data were uploaded to a secure portal and double-scored independently per AASM criteria.

**Results:**

Of the 41 home sleep studies, 83% were of sufficient quality for diagnosis by an expert assessment, and 87.8% met rule-based success criteria. Signal quality was high, with ~80% of each signal providing over 5 hours of scorable data. Interscorer agreement was excellent for all sleep parameters, with the highest agreement in AI (0.98), AHI (0.97), REM (0.95) and OAI (0.95). Participant usability and satisfaction was high, 87.8% experienced no problems with device application, 98.1% found the self-application time acceptable, and 90.7% indicated they would recommend the device.

**Conclusions:**

The patch-based home PSG device demonstrated high success rates with excellent signal quality, high interscorer agreement, and overall high participant satisfaction. The device appears to be a viable alternative to in-lab PSG, offering a user-friendly solution for comprehensive sleep assessment in a home setting.

Registration. The observational study described in this manuscript was retrospectively registered with an internationally recognized trial registry (ClinicalTrials.gov, URL: https://clinicaltrials.gov/study/NCT06881667, Name: " US Development and Evaluation Study of a Patch-Based PSG system”, ID: NCT06881667, Date: February 26th, 2025).

## Introduction

There are over 80 sleep disorders that impact sufferers’ health and well-being, leading to issues with daytime functioning, quality of life, mental health, and an increased risk of comorbid chronic disease [1–5]. As the population continues to age and obesity rates rise, the prevalence of all sleep disorders is expected to increase at a rate of 12.3% between 2024 and 2032 [6].

There are two types of diagnostic studies for sleep disorders used in clinical practice. Home Sleep Apnea Tests (HSATs) are a low-cost test utilized in patients with a high probability of moderate-severe obstructive sleep apnea (OSA) and minimal comorbidities. While HSATs are convenient, scalable and cost-effective, they lack the comprehensive physiological data needed to detect neurological sleep disorders, rely on an indirect measure of sleep and clinical guidelines limit their use to patients with no significant comorbidities [7]. Polysomnography (PSG) has an expanded signal set that facilitates its use in the diagnosis of neurological sleep disorders, respiratory sleep disorders in patients with comorbidities, and patients with respiratory disorders with a failed HSAT [7]. Polysomnography is the gold standard measurement for all sleep disorders and cannot be substituted with HSATs in all clinical cases.

Sleep centers cannot meet current demand for in-lab PSG studies due to limitations in bed capacity and trained personnel. Additionally, a 12.3% increase in demand for in-lab PSG studies will place an unprecedented cost burden on public and private payers. Alternative diagnostic methods that can address infrastructure limitations at a lower cost may provide an acceptable solution.

The evidence to date suggests that level 2 sleep devices, capable of unattended home polysomnography, provide diagnostic accuracy comparable to in-lab studies [8]. Emerging data further support self-applied devices, showing that with clear instructions and user-friendly designs, patients can achieve reliable recordings without significant loss of data quality [9, 10]. These approaches hold promise as scalable, cost-effective, and patient-centered diagnostic tools. The Onera Sleep Test System (STS) 1 is a first-generation, patch-based, type-II PSG device designed for self-applied, at-home PSG studies. The Onera STS 1 offers a 15-channel signal set acquired through four easy-to-apply patches on the head, chest, abdomen, and leg. This wireless, patch-based design prioritizes patient comfort and ease of application, eliminating the need for a technician to travel to the home for device setup. The Onera STS 1 has been technically validated against in-lab PSG in a large (*N* = 206), technician-applied, side-by-side clinical trial for sleep staging and respiratory events [11]. The next step in device evaluation is to use the patch-based device in the real-world use case self-applied by patients, at home. In this observational study, the success rate, feasibility and acceptance of self-applied home PSG studies using the patch-based system was investigated.

## Methods

### Participants

Consecutive adult patients referred for a sleep study at two clinical sites, (1) Clayton Sleep Institute, St. Louis, Missouri, and (2) The Sleep Disorders Center of Alabama, Birmingham, Alabama, were included in the study if they had no contraindications to the patch-based PSG device and consented to participate. Clayton Sleep Institute has emerged as a tertiary referral center, where patients who have been previously diagnosed but struggle with current therapy or remain symptomatic are routinely referred to. Study recruitment ran from August 2023 to September 2024.

### Home PSG Study

Participants were introduced to the patch-based PSG and instructed to review the manual at home. Participants were sized for the head patch by the study staff and shown how to position the head patch for application.

Within one week, participants received the patch-based PSG device by mail. The package included an instructional leaflet, a QR code linking to video instructions, and a phone number for device support. Participants were encouraged to perform their sleep study within one week of receiving their device.

On the morning after the participant’s sleep study, a research coordinator from the site contacted the participant over the phone to complete a survey about their experience with the patch-based PSG device. The survey included questions about device usability with “Yes” and “No” questions and the opportunity to expand on the answer in a freeform response, and about the satisfaction with the patch-based device on a Likert scale.

After completing the home sleep study, the participant shipped the device back to the fulfilment center in the supplied, pre-paid packaging. The fulfilment center uploaded the participant’s data to a secure cloud environment and sent it for blind scoring according to the AASM 2020 guidelines V2.6 (using the 3% or arousal hypopnea rule) to two scorers: Scorer (1) Sleep Scoring Services LLC and Scorer (2) an AASM accredited sleep scorer [12].

### Device: The Onera STS

**Fig. 1:**
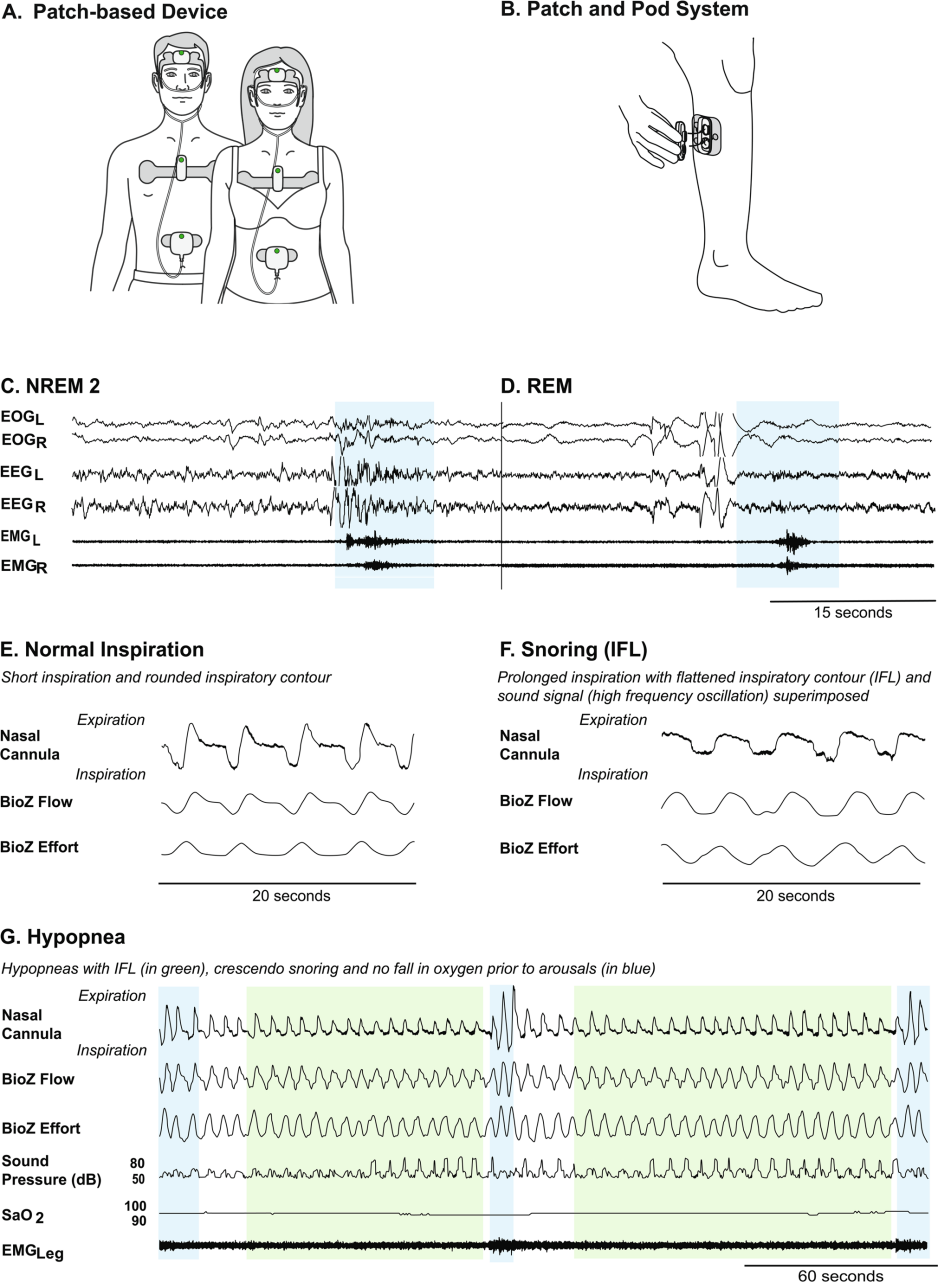
The patch-based, type-II PSG system consists of 4 sensors located on the head, chest, abdomen (**A**) and leg (**B**). The patch-based sensors are comprised of disposable patches and reusable pods that snap into the patches for data collection (**B**). Example signals collected from the head sensor used to score sleep can be seen in (**C**) NREM2, and (**D**) REM. Signals highlighted in blue are arousal events during sleep. Different respiratory events collected via the chest sensor and SpO2 sensor can be seen, such as Normal Inspiration (**E**), Snoring (Inspiratory Flow Limitation (IFL)) (**F**) and Hypopneas (**G**). Signals highlighted in green are hypopnea events

The Onera STS 1 (legal manufacturer Onera B.V., Eindhoven, Netherlands) is a wearable patch-based, type-II PSG system for measuring physiological signals during a sleep study conducted at home [13]. The patch-based PSG system consists of 4 disposable patches self-applied to the forehead, upper chest, abdomen, and lower leg, and reusable pods that snap into the patches to measure the physiological signals required for sleep study, see Fig. 1. The head sensor (patch and pod system combined) measures electroencephalography (EEG) (Frontal, Left and Right), electrooculography (EOG) (Left and Right), electromyography (EMG) masseter (Left and Right) and Oxygen Saturation (SpO2). The chest sensor measures electrocardiography (ECG), respiratory flow based on a bio-impedance based measurement (BioZ flow), respiratory effort based on a bio-impedance based measurement (BioZ effort), activity (using an accelerometer), sound pressure level (via a chest microphone) and body position (using an accelerometer at the chest). The flow sensor measures nasal pressure using a nasal cannula. The leg sensor measures EMG from the anterior tibialis muscle.

A sleep study completed with the Onera STS is uploaded to the secure cloud platform in the form of an Electronic Data File (EDF). The Onera STS EDF can be scored using any scoring software to generate a PSG report.

### Inclusion Criteria

- 18 years and older.
- Referral for a suspected sleep disorder requiring a sleep diagnostic study.

### Exclusion Criteria

- A known history of allergic reactions to adhesives or hydrogels, or a family history of adhesive skin allergies.
- Severe skin conditions at the site of patch application such as wounds, burns, or damaged skin.
- An implanted cardiac stimulator or diaphragmatic pacer.
- Expected exposure to high-frequency surgical equipment, strong magnetic fields, or external cardiac stimulators during the use of the patch-based PSG device.

- Inability to provide informed consent.
- Has an anatomical abnormality that, in the opinion of the Principal Investigator, makes the subject ineligible for inclusion.

### Study Analysis

PSG studies were assessed using two methods: (1) expert assessment and (2) rule-based assessment.

#### Expert Assessment

A study investigator reviewed each sleep study to determine whether it was adequate for a clinical diagnosis.

#### Rule-Based Assessment

Artifact free signal quantity was calculated for each sleep study and a study rating was given based on rule-based criteria, see Table 1. A rating of Fair or greater denoted a successful sleep study.

**Table 1:** Rule-based assessment criteria based on the availability and quality of a signal set. An * indicates a failed sleep study

Rating	Criteria
Outstanding	All channels (EEG, EOG, EMG masseter, oximetry, nasal pressure, BioZ effort) good for ≥ 6 h.
Excellent	At least one EEG channel, one EOG channel, one masseter EMG channel, oximetry, nasal cannula flow, BioZ Effort good for ≥ 5 h.
Very Good	At least one EEG channel, oximetry, nasal cannula flow and BioZ effort good for ≥ 4 h.
Good	Respiratory channels (nasal cannula or BioZ effort), oximetry and one EEG good for ≥ 3 h.
Fair	Respiratory channels (nasal cannula airflow or BioZ effort), oximetry and one EEG good for ≥ 3 h < 4 h. EEG signal must be good enough to determine sleep from wake.
Poor*	Respiratory channels (nasal cannula airflow and BioZ effort), oximetry signals, or EEG channels contain < 3 h of data, but interpretable data on any other channel.
Unsatisfactory*	No usable data. Less than 2 h on all channels

### Statistical Methods

Sleep summary statistics were calculated using mean and standard deviation. Participant survey answers were summarized on a Likert scale or a pie chart where appropriate. The intraclass correlation coefficient (ICC) was implemented using the python pingouin statistical package, version 0.5.5, based on a mean-rating (k = 2), absolute, 2-way mixed effects model [14]. ICC was evaluated using the following guideline [15]. All analysis was completed using python. No imputation was made for missing values.

## Results

### Participants

Of the 42 participants who consented, 1 participant cancelled the sleep study, and 41 participants completed a home patch-based PSG study.

### Demographics

The demographics of all participants who completed a home, patch-based PSG study can be found in Table 2. No statistical differences were found between participants that had successful and unsuccessful sleep studies using both the expert and rule-based assessments.

**Table 2:** Demographics for 41 participants who completed a sleep study. Data is presented as N (%) or Mean ± SD

Demographics	All Participants (*N* = 41)
Age	48.7 ± 16.9
> 65	9 (22.0%)
BMI Distribution	
BMI	32.3 ± 7.4
Normal Weight	6 (14.6%)
Overweight	10 (24.4%)
Obesity I	13 (31.7%)
Obesity II	7 (17.1%)
Obesity III	5 (12.2%)
Sex	
Male	24 (58.5%)
Female	17 (41.5%)
Race	
Black	3 (7.3%)
White	37 (90.2%)
Asian	1 (2.4%)
Ethnicity	
Not Hispanic or Latino	41 (100.0%)
Education Level	
High School	5 (12.2%)
< Bachelor’s Degree	12 (29.3%)
Bachelor’s Degree	16 (39.0%)
>Bachelor’s Degree	8 (19.5%)
Technological Comfort	
Uncomfortable	1 (2.4%)
Neutral	5 (12.2%)
Comfortable	17 (41.5%)
Very Comfortable	18 (43.9%)
Suspected Sleep Disorder	
Obstructive Sleep Apnea	34 (82.9%)
Unreported	7 (17.1%)
Comorbidities	
Depression	6 (14.6%)
Diabetes	5 (12.2%)
High Cholesterol	10 (24.4%)
Hypertension	11 (26.8%)
Thyroid Diseases	3 (7.3%)

### Expert Assessment

#### Success Rate

The investigator concluded that 34 of the 41 studies were of sufficient duration and quality to lead to a sleep diagnosis, a success rate of 83%.

### Rule-based Assessment

#### Signal Assessment

For all signals, except for EEG R, 80% of studies had greater than 5 h of artifact free signal data, see Table 3. EEG R had 78% of studies with more than 5 h of artifact free signal quality. The signals with the greatest proportion of studies with < 3 h of artifact free data were EEG R, EOG L, EMG masseter L, EMG masseter R and SpO2, all at 12.2%. This is consistent with the sleep study success rate that is seen in Table 4.

**Table 3:** Results from the artifact-free signal quantification portray the percentage of studies with signal quantity and quality above a threshold (hours)

	Head Sensor							Chest Sensor		Flow Sensor		Leg Sensor
Hours	EEG L (%)	EEG R (%)	EOG R (%)	EOG L (%)	EMG masseter L (%)	EMG masseter R (%)	SPO2 (%)	ECG (%)	Bio Z Effort (%)		Nasal Cannula Flow (%)	EMG Leg (%)
≥ 6.0	73.2	70.7	78	82.9	85.4	82.9	73.2	92.7	78		82.9	87.8
5.0–5.9	7.3	7.3	9.8	4.9	2.4	2.4	7.3	2.4	4.9		4.9	4.9
4.0–4.9	7.3	7.3	0	0	0	0	4.9	0	2.4		0	2.4
3.0–3.9	4.9	2.4	2.4	0	0	2.4	2.4	0	4.9		2.4	2.4
< 3.0	7.3	12.2	9.8	12.2	12.2	12.2	12.2	4.9	9.8		9.8	2.4

**Table 4:** Study ratings as given by rule-based criteria outlined in Table 1. * Indicates a failed sleep study

Rating	Rule-based Assessment
Outstanding	19 (46.3%)
Excellent	4 (9.8%)
Very Good	3 (7.3%)
Good	9 (22.0%)
Fair	1 (2.4%)
Poor*	4 (9.8%)
Unsatisfactory*	1 (2.4%)

#### Success Rate

Success rate calculated using the rule-based success criteria was 87.8%, see Table 4. 56.1% of studies showed Outstanding or Excellent results, 29.3% of studies had Very Good or Good Results and 2.4% of studies were rated Fair. Ultimately, 12.2% of studies were Poor or Unsatisfactory, not meeting the minimum rule-based criteria for a successful sleep study.

### Inter-scorer Agreement

Sleep summary statistics and interscorer agreement can be found in Table 5. Overall, the interscorer agreement assessed using ICC was excellent (≥ 0.80) for most sleep variables. The highest agreement between sleep scorers was Apnea Index (AI) (0.98), followed by Apnea-Hypopnea Index (AHI) (0.97), Rapid Eye Movement (REM) (0.95) and Obstructive Apnea Index (OAI) (0.95). The lowest agreement between sleep scorers was for the Arousal Index (0.62).

**Table 5:** 41 sleep studies were scored by two independent, certified scorers. The sleep summary statistics are presented using mean ± standard deviation. The interscorer agreement was evaluated using intra-class correlation (ICC)

Variable	Scorer 1	Scorer 2	Interscorer Agreement (ICC)
TST	419.63 ± 87.74	396.08 ± 88.20	0.94
NREM	321.48 ± 57.04	314.24 ± 67.49	0.93
N1	17.47 ± 11.96	34.38 ± 17.92	0.32
N2	279.81 ± 57.95	248.16 ± 69.99	0.86
N3	24.20 ± 23.90	34.48 ± 32.02	0.83
REM	76.10 ± 41.63	81.85 ± 38.03	0.95
AHI	17.22 ± 19.78	15.90 ± 18.67	0.97
AHI REM	22.97 ± 22.60	21.58 ± 23.23	0.92
HI	11.03 ± 9.54	7.68 ± 7.37	0.86
AI	6.18 ± 15.07	8.23 ± 15.31	0.98
CAI	1.28 ± 4.30	0.72 ± 1.40	0.64
OAI	4.90 ± 11.88	7.51 ± 14.59	0.95
Arousal Index	3.45 ± 3.84	3.58 ± 4.10	0.62
ODI	19.70 ± 21.73	14.70 ± 20.45	0.93

### Participant Perception of the Patch-Based Device

#### Device Usability Questionnaire

**Fig. 2:**
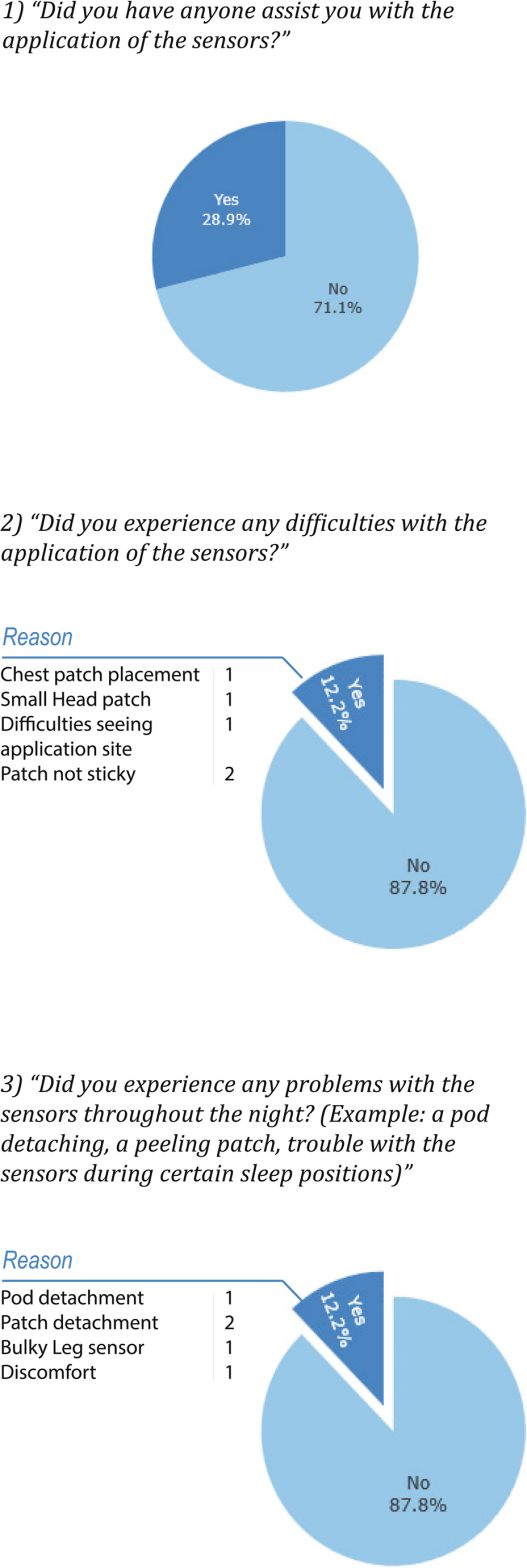
Results from the usability questionnaire after completion of a home sleep study. Results are presented as percentages where participants were asked to elaborate for “Yes” answers.

28.9% of participants sought assistance in device application from a household member, see Fig. 2. Additionally, 12.2% reported application difficulties and problems throughout the night.

#### Participant Satisfaction Questionnaire

**Fig. 3:**
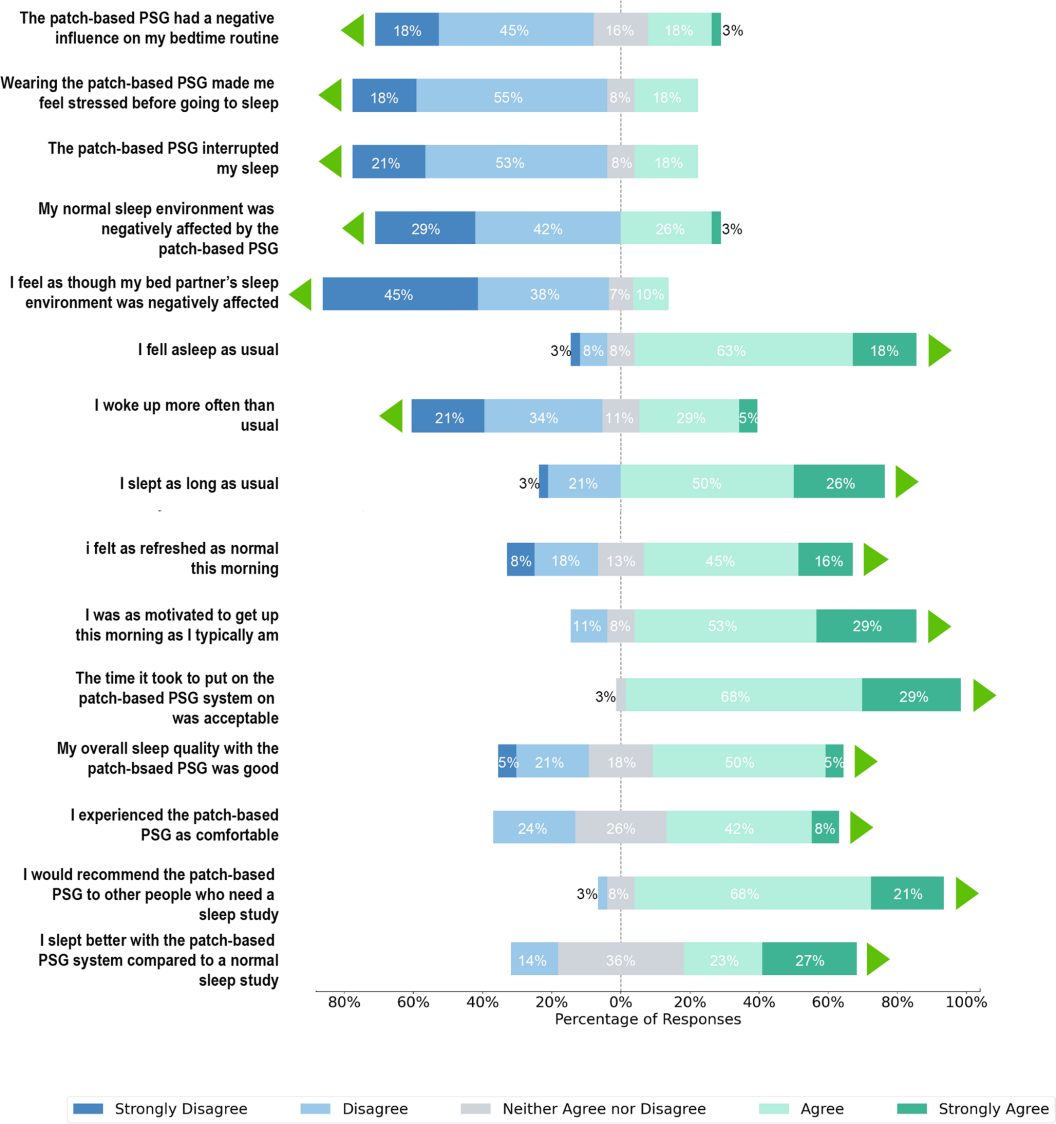
Results from the participant satisfaction questionnaire after completion of a home sleep study. Questions were asked on a Likert scale and the results are presented as the percentage of each response. The green triangles in the figure indicate the direction of a favorable response to the patch-based device

Participant responses to a satisfaction questionnaire, Fig. 3, are summarized in the text below. Endorsing the statement indicates that participants responded “Agree” or “Strongly Agree” and not endorsing the statement responded “Disagree” or “Strongly Disagree”.

##### Bedtime routine and sleep environment

63% did not endorse the statement that the patch-based device had a negative influence on their bedtime routine and 71% did not have a negative influence on their usual sleep environment. Further, 83% did not endorse the statement that their partner’s sleep environment was adversely affected.

##### Sleep characteristics

74% of participants did not endorse the statement that the Onera System interrupted their sleep. 81% endorsed falling asleep as usual and 76% endorsed sleeping for as long as they normally would. 55% of participants did not endorse waking more often than usual.

##### Next day

61% endorsed feeling as refreshed as normal upon waking and 82% endorsed feeling as motivated to get up as they typically would.

##### Satisfaction

97% endorsed that the time required to put on the patch-based device was acceptable. 55% endorsed that their overall sleep quality was good while using the system. 50% endorsed that the patch-based device was comfortable. 89% endorsed that they would recommend the patch-based PSG to others.

##### Previous sleep study experience

Of those who had had a previous sleep study (61% of all participants), 50% endorsed that they slept better using the patch-based device than a previous sleep study.

### Adverse Events

A total of 6 Adverse Events (AEs) (14.6%) related to the Onera STS device were reported. Five events (12.2%) were classified as mild- skin itchiness or redness- and resolved within one week. One event (2.4%) was deemed to be a moderate AE, a skin abrasion lasting 2 weeks. There were no serious or severe adverse events. All AEs resolved without clinical sequalae.

## Discussion

Our findings demonstrate that participants achieved a high study success rate using the self-applied, patch-based PSG and report strong acceptance of the device and study process.

Challenges exist in the comparison of home PSG success rates. A review of the literature by Braun et al. revealed considerable variation in how home PSG studies were conducted and evaluated [8]. Out of 30 studies included in this review, only 4 studies completed home PSG studies in the home environment, self-applied by the participant, and of those 4, only 2 are published papers [16, 17].

The two published articles use success criteria proposed by Redline et al. and Punjabi et al. [16, 18]. Redline’s criteria evaluate PSG signal integrity and redundancy, requiring a minimum of four hours of acceptable signal quality for a passing study rating. Although thorough, Redline’s criteria were developed and most widely used for technician-applied home PSG studies, therefore not suitable for benchmarking against our data. Punjabi proposed alternative success criteria for self-applied home studies using the Nox A1 device in which he lowered the minimum recording time to 3 h for all signals. Although the study was similarly self-applied in the home environment, Punjabi did not account for signal redundancy in his criteria development.

The rule-based assessment criteria proposed in our analysis combined pertinent elements from both the Redline and Punjabi criteria, adopting Redline’s signal evaluation method and consideration for signal redundancy and Punjabi’s, three-hour threshold for self-applied devices. When using the rule-based criteria, we reported a patch-based PSG study success rate of 88%. When benchmarking against only the self-applied, at-home type II PSG studies from the Braun review and a recently published manuscript, we found similar reported success rates of 84% (Punjabi criteria), 95% (Redline criteria) and 84.5% (success criteria not stated) [10, 16, 17].

Rule-based success criteria are artificially derived research tools that provide an estimate for what is a clinically acceptable sleep study. There are clinical scenarios in which study grading by rule-based criteria might falsely accept a clinical study or reject a study despite robust clinical evidence of a diagnosis. For example:

- a patient completes a sleep study recording with a failed effort signal but has a high-resolution nasal cannula airflow signal depicting upper airway obstruction, such as in Fig. 1 (E, F,G).
- Total recording time is 3 h but the patient has severe sleep apnea.
- Total recording time is 3 h and there are visible body movements during REM sleep indicative of REM behavior disorder.

The clinical cases above would have been rejected by either the Redline or Punjabi criteria, however, a physician would likely have sufficient data for a clinical diagnosis. As such, we believe there is a need to balance rule-based success criteria with clinical judgement when making decisions on a home PSG study’s acceptance.

Accordingly, we asked the investigator to perform an expert assessment as a second measure of study success. The expert assessment of the patch-based PSG studies resulted in a success rate of 83% (vs. 88% Rule-based assessment). As far as we are aware, we are one of few publications to validate our rule-based assessment criteria against an expert for clinical use.

Patient acceptance and ease of use are crucial determinants of a successful home PSG study. Results from our sleep study survey observed that the majority of participants found the application straightforward, that device acceptance and comfort were high, and that the patch-based device minimally disrupted bedtime routines and sleep quality. Participants also noted no adverse effects on their bedroom environment or their next day state and many expressed willingness to recommend the device to others. Despite 1/3 of patients asking for a household member’s assistance, the majority of participants considered the device user-friendly, as reflected in the high study success rates in both the rule-based and expert assessments.

In addition to patient acceptance, a device must provide reliable results to gain clinical acceptance. The data revealed that almost 80% of studies had greater than 5 h of high signal quality per signal and interscorer agreement was shown to be above 0.8 for almost all metrics. The low interscorer agreement in N1 was an unsurprising find and is well documented in literature [19, 20]. The results suggest that the patch-based PSG produces high quality signals that can be reliably scored according to AASM criteria.

Sleep centers are already struggling to meet current demand for in-lab PSG due to bed and trained personnel shortages, leading to long waiting times for patients. A new diagnostic device that facilitates home PSG studies must reduce the burden on clinical staff and seamlessly integrate into clinical workflow. Home PSG devices that require a technician to travel to the participant’s home or multiple night studies have the potential to add new bottlenecks to the diagnostic process. In contrast, the results of this observational study demonstrated that with minimal device introduction, a successful self-applied, at-home study can be completed using the patch-based PSG device.

Reimbursement is a critical factor for clinical adoption of the home PSG category. Home PSG is formally reimbursed in the Netherlands and Austria, and partially reimbursed in Germany, France, and Canada. Securing a dedicated CPT code with insurance coverage and payment will be essential to drive broader adoption of home PSG in the US.

A few limitations in the clinical study are important to mention. Firstly, the majority of participants were highly educated (≥ bachelor’s degree) and reported that they were comfortable or very comfortable with technology. Although patients were consecutively referred to participate in the study there may be a center or participation self-selection bias. Secondly, expert and rule-based study assessments were each performed by a single evaluator. Although this is common in clinical practice, Redline and Punjabis grading criteria likewise used a single rater, reliance on one individual can introduce bias [16, 18]. Thirdly, we have not yet measured the acceptance rate of the patch-based PSG in a real-world, large-scale clinical setting across the patient spectrum as most participants in this clinical study were referred on the basis of suspected OSA. Future publications will include real-world success rates across multiple clinics currently using the patch-based PSG device in their routine practice.

## Conclusion

In summary, the patch-based PSG provides high-quality sleep data for a high study success rate while demonstrating high user-acceptance and minimally disrupting clinical workflows. While existing rule-based guidelines remain valuable tools for estimating success rates, our experience underscores the importance of clinical context and judgment. By balancing these considerations, the Onera STS 1 shows promise as an effective, convenient, and participant-friendly solution for home-based sleep diagnostics.

## Data Availability

Data from this research is not available to share to protect study participant privacy.
